# Vesicoureteric reflux: Evaluation by bladder volume graded direct radionuclide cystogram

**DOI:** 10.4103/0971-9261.45360

**Published:** 2009

**Authors:** Vikesh Agrawal, Venkatesh Rangarajan, Tejaswini Kamath, S. S. Borwankar

**Affiliations:** Department of Pediatric Surgery, Seth G S Medical College and KEM Hospital, Mumbai, India

**Keywords:** Bladder volume, children, direct radionuclide cystogram, vesicoureteric reflux

## Abstract

**Aim:**

Evaluation of vesicoureteric reflux (VUR) in children by bladder volume graded direct radionuclide cystogram (BVG DRC). This technique allows detection of VUR at different bladder volume grades.

**Materials and Methods:**

In this prospective study, 33 patients (66 renal units) with suspected vesicoureteric reflux were subjected to a voiding cystourethrogram (VCUG) and BVG DRC. The patients were assessed further with radioisotope renal scans for renal cortical scars.

**Results:**

Twenty-two patients and 36 renal units were found to have VUR in either of the reflux studies. A VCUG was able to detect 20 units (55.50%) and a BVG DRC was able to detect 35 units (97.2%). A VCUG had a test accuracy of 77.8% and a BVG DRC had a test accuracy of 98.6%. There was a positive correlation between bladder volume grades and scarring on a DMSA scan.

**Conclusions:**

Like a conventional DRC, BVG DRC is a sensitive and an accurate test. It gives additional information on the reflux phenomenon with respect to bladder filling. The bladder volume graded technique is better than conventional DRC for grading of VUR.

## INTRODUCTION

The potential, harmful consequences to the kidneys due to vesicoureteric reflux suggest the need for early detection, grading, and management.[1] Voiding cystourethrography (VCUG) is the gold standard of detecting and characterizing reflux. If VCUG is done with continuous fluoroscopy radiation, the burden is significantly increased.[2] Direct radionuclide cystography (DRC) is associated with a higher sensitivity to detect reflux, minimal radiation exposure, and can detect intermittent reflux, which might be missed during a VCUG done with spot films or with intermittent or no fluoroscopy at all.[3]

Intravesical pressure and bladder physiology attribute important factors in the occurrence of reflux. Reflux at high bladder pressures can often occur even in the presence of low bladder volumes. Reflux at a low bladder volume is likely to be more frequent, is associated with a resistance to spontaneous resolution, and hence more severe.[4,5] Bladder volume graded direct radionuclide cystogram (BVG DRC) is a technique that allows the detection of reflux at different bladder volumes during the filling phase and during voiding. BVG DRC incorporates the advantages of conventional DRC and significantly low radiation burden. Plenty of literature is available on conventional DRCs while very few studies have established the role of bladder volume assessment concurrently with reflux phenomenon.[6,7] We studied the utility of bladder volume graded technique of DRC in the evaluation of vesicoureteric reflux.

## MATERIALS AND METHODS

This is a prospective institution-based study carried out at Seth G.S. Medical College (KEM and BJ Wadia Children Hospitals) and Radiation Medicine Center, Tata Memorial Hospital. A total of 33 patients of either gender less than 12 years of age with suspected VUR were included.

The patients included in the study were new and antenatally diagnosed with hydroureteronephrosis, bilateral hydronephrosis, suspected posterior urethral valve, patients with proven urinary tract infection (UTI), cases of neurogenic bladder secondary to meningomyelocele above 1 years old, and anorectal malformation with hydronephrosis or documented UTI. Patients with unilateral hydronephrosis without hydroureter and anorectal malformation without hydronephrosis were excluded from the study.

All patients were subjected to VCUG and BVG DRC in the same week. All patients underwent Diethylene triamine pentaacetic acid (DTPA) and Dimercaptosuccinic acid (DMSA) renograms. As only an intermittent fluoroscopy was used for VCUG, the volumes of bladder at which reflux occurred could not be recorded. A BVG DRC was done at the Radiation Medicine Center, Tata Memorial Hospital by a pediatric nuclear physician, pediatric surgery senior resident, and nuclear medicine technician.

**Figure 1:**
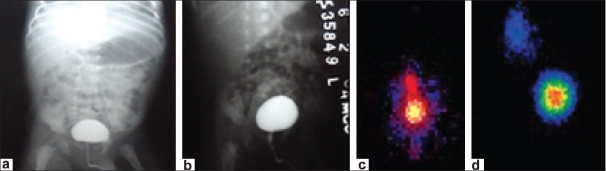
(a) Filling phase VCUG showing no reflux; (b) Voiding phase VCUG showing no reflux; (c) BVG-DRC, filling phase, severe grade reflux on right side (d) BVG-DRC, voiding phase, severe grade reflux on right side

**Figure 2:**
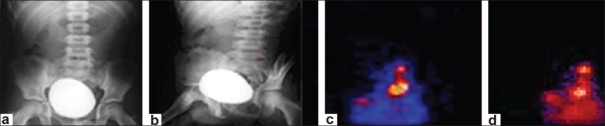
(a) Filling phase VCUG showing no reflux; (b) Voiding phase VCUG showing no reflux; (c) BVG-DRC, filling phase, severe grade reflux on left side at low bladder volume; (d) BVG-DRC, voiding phase, severe grade reflux on left side

The technique of BVG DRC was as follows: The bladder was emptied prior to the study. The dynamic acquisition starts for the filling phase with infusion of 0.5mCi of 99mTcO4 into the bladder. The filling of saline was at the rate of approximately 10% of the bladder capacity for every infusion until the amount finishes until the maximum calculated capacity was reached as per Koff's formula. A record of the volume of saline at which reflux was first seen was noted along with the conventional radionuclide grade. Three images were taken: A 30-seconds anterior prevoid static image, a voiding dynamic, and an anterior post-void 30 seconds static image. Acquisition was done at two frames per second for the filling and voiding phase. Conventional DRC radionuclide grading was as follows: Ureter only-mild, ureter and pelvis/ undilated-moderate, and ureter and pelvis/dilated-severe.[1] The grading of reflux is done in relation to estimates of bladder filling; the technique used is called bladder volume graded DRC.[6,7] A low reading is considered if reflux is first evident on the first third bladder filling, a high reading is on the final third of maximum bladder volume, and a moderate reading is in between. Reflux with voiding is also noted (Figures 1 and 2 show the representative studies in two different patients with both having reflux on right and left side respectively). All the VCUG plates were reviewed and graded according to the international classification for grading of vesicoureteric reflux.

A statistical analysis was done on Statistical Programming for Social Sciences (SPSS, Version 11.5) and STATISTCA, Version 5.5; *P-* value < 0.05 was considered statistically significant.

## RESULTS

A total of 33 patients were subjected to a bladder volume graded direct radionuclide cystogram; the distribution is shown in Table 1. There were 30 males and 3 females. The age of the patients ranged from Day 1 of life to 11 years old. Overall, 66 renal units were examined for the presence and characteristics of vesicoureteric reflux.

**Table 1:** Etiology of vesicoureteric reflux (total no=22)

Diagnosis	n (%)
Primary VUR	10 (45.45)
Posterior urethral valves	8 (36.36)
Neurogenic bladder with meningomyelocele	1 (4.54)
Anorectal malformation	1 (4.54)
Pelviureteric junction obstruction	1 (4.54)
Duplex renal system	1(4.54)

VUR: vesicoureteric reflux

Twenty-two patients and 36 out of 66 renal units (54.5%) were found to have VUR on the reflux study. The mean age in the refluxing group of patients was 3.22±3.71 years. The VCUG was falsely negative in 16 units (44.44%) while BVG DRC was falsely negative in 1 unit (2.77%). Overall sensitivity of BVG DRC and VCUG were 97.2% and 55.6%, respectively. Both tests were 100% specific when confirmed on cystoscopy. VCUG had a test accuracy of 77.8% (0.778 ± 0.058, *P* < 0.0001) and BVG DRC had test accuracy of 98.6% (0.986 ± 0.016, *P* < 0.0001).

When the refluxing units were distributed according to bladder volume grades, most of the units with VUR were detected to have VUR during the voiding phase (97.4%). While the detection rate during low bladder volume grade, moderate bladder volume grade, and high bladder volume grade were 42.8%, 54.2%, and 74.2%, respectively. Out of 36 refluxing renal units, 24 units (66.6%) had evidence of renal scarring on a DMSA scan. Scarring was most visible in low bladder volume grade (93.33%; 14 out of 15 renal units), 60% for moderate grade (3 out of 5 renal units), 42.85% for severe grade (3 out of 7 renal units), and minimum (25%) in voiding (2 out of 8 renal units). *P* = 0.0029 was statistically significant for LBVG vs. voiding grades.

There was positive correlation between bladder volume grades and scarring (Pearson's correlation coefficient, +0.582). The Pearson's correlation coefficient between VCUG and conventional DRC grades with scarring was +0.457 and +0.468, respectively. The cut-off value for a statistically significant high occurrence of scarring for VCUG was ≥ Grade 3 while it was High bladder volume grade of BVG-DRC ie., all reflux occurred during last one-third of bladder filling phase (*P* < 0.005).

## DISCUSSION

VUR may be primary or secondary. The severity of reflux is the most important factor in determining the outcome of disease. The spontaneous resolution rates are 92-100% in Grade I, 63-76% in Grade II, 53-62% in Grade III, and 32-33% in Grade IV.[8]

The disadvantage of the conventional DRC is the inability to grade reflux accurately due to the high level of radioactivity in the bladder and scatter of the gamma photons. Also, the radionuclide grading of reflux is highly observer-dependent and depends on the dilatation, tortuosity, and the level of tracer activity in the system.[3] Reflux occurring at the early filling phase and low bladder volumes is likely to be more frequent and severe. A bladder with decreased compliance certainly places the upper tracts at risk of damage.[5] This provoked us to find a test for detection of reflux with high sensitivity and an objective grading of reflux that has more prognostic value.

McLaren and Simpson have shown that grading of reflux by DRC at different bladder volumes provides additional information and has been called bladder volume graded direct radionuclide cystogram.[6,7] This study is the second study to evaluate this technique in the evaluation of VUR. The accuracy of the BVG DRC is better than that of the VCUG, which is supported by this study. The severity of reflux is conventionally measured by VCUG or DRC, but none of them grade the reflux with bladder volumes. This study highlights the importance of the assessment of volume grade of bladder at which reflux occurs. It neither tries to endorse nor contradict the already established features and roles of VCUG or conventional DRC.[9–11]

A total of 83% of Grade 1 reflux (5 out of 6 renal units) according to conventional DRC grading identified at low bladder volume grade had evidence of renal scarring. This suggests the fact that even mild grade reflux of conventional VCUG or DRC grade can be a threat for renal scarring, if identified at a low bladder volume grade. It is this mild grade reflux that is often missed on VCUGs and has clinical value if the BVG DRC grading system is followed. The severity of renal scarring is directly related to the grade of vesicoureteric reflux.[12] This study highlights the importance of this fact by the following results: The VCUG missed 8 refluxing renal units with scars, high correlation of scarring with conventional and bladder volume grades, and high incidence of scarring at low bladder volume grades on BVG DRC.

In this study, 16 patients who were older than 1 year old underwent an urodynamic study. Six patients (37.5%) were identified as having high pressure and poor compliance. All of the patients had severe reflux on VCUG and conventional DRC grading and reflux during low bladder volume on BVG DRC, suggesting a correlation of high bladder pressures with a grade of reflux. Both primary and secondary VUR are included in the study, although they behave differently as far as outcome is concerned. But the bladder volume plays an important role in the etiology and pathogenesis of both the conditions.[4,5]

In the present study, it was difficult to determine the false positive DRC results, but every endeavor was made to identify technical artifacts that may give rise to false-positive DRC results. The same volume grading protocols can be applied to VCUG only if continuous fluoroscopy is used, which increases radiation hazards.

The technique has all the disadvantages that conventional DRC has, such as failure to identify bladder and urethral morphology and less cost effectiveness. The severity of reflux is conventionally measured by VCUG or a conventional DRC, but none of them grade the reflux with respect to bladder volumes. This study highlights the importance of assessment of the volume grade of the bladder at which reflux occurs. Long-term follow-up is being to done to further evaluate the outcome of active intervention based on the results of this study.

## CONCLUSIONS

Bladder volume graded direct nuclide cystography is a better grading system for VUR. It has all the advantages of conventional DRC with the added advantage of assessment of VUR in terms of bladder physiology. Bladder volume grading is concurrently possible with conventional DRC grading and should be done in all DRCs. The reflux encountered at low bladder volumes are more harmful and associated with a higher risk of renal scarring, so it should be identified at the onset. Bladder volume graded DRC provides a better investigation to prognosticate the disease for renal damage and will be more useful in planning the management of VUR.
